# Neurobiology of Risk for Bipolar Disorder

**DOI:** 10.1007/s40501-016-0093-6

**Published:** 2016-10-20

**Authors:** Ayşegül Özerdem, Deniz Ceylan, Güneş Can

**Affiliations:** 1 Department of Psychiatry, Faculty of Medicine, Dokuz Eylül University, Izmir, Turkey; 2 Department of Neuroscience, Health Sciences Institute, Dokuz Eylül University, Izmir, Turkey; 3 Department of Psychiatry, Gümüşhane State Hospital, Gümüşhane, Turkey

**Keywords:** Bipolar disorder, Endophenotype, Risk, Relatives, Neurocognition, Brain imaging, Oxidative stress

## Abstract

Bipolar disorder (BD) is a chronic mental illness which follows a relapsing and remitting course and requires lifetime treatment. The lack of biological markers for BD is a major difficulty in clinical practice. Exploring multiple endophenotypes to fit in multivariate genetic models for BD is an important element in the process of finding tools to facilitate early diagnosis, early intervention, prevention of new episodes, and follow-up of treatment response in BD. Reviewing of studies on neuroimaging, neurocognition, and biochemical parameters in populations with high genetic risk for the illness can yield an integrative perspective on the neurobiology of risk for BD. The most up-to-date data reveals consistent deficits in executive function, response inhibition, verbal memory/learning, verbal fluency, and processing speed in risk groups for BD. Functional magnetic resonance imaging (fMRI) studies report alterations in the activity of the inferior frontal gyrus, medial prefrontal cortex, and limbic areas, particularly in the amygdala in unaffected first-degree relatives (FDR) of BD compared to healthy controls. Risk groups for BD also present altered immune and neurochemical modulation. Despite inconsistencies, accumulating data reveals cognitive and imaging markers for risk and to a less extent resilience of BD. Findings on neural modulation markers are preliminary and require further studies. Although the knowledge on the neurobiology of risk for BD has been inadequate to provide benefits for clinical practice, further studies on structural and functional changes in the brain, neurocognitive functioning, and neurochemical modulation have a potential to reveal biomarkers for risk and resilience for BD. Multimodal, multicenter, population-based studies with large sample size allowing for homogeneous subgroup analyses will immensely contribute to the elucidation of biological markers for risk for BD in an integrative model.

## Introduction

Bipolar disorder (BD) is a chronic mental illness which follows a relapsing and remitting course and requires lifetime treatment. In nearly two thirds of patients, the illness begins before the end of the third decade of life [1]. BD is heritable as shown by varying (59–93 %) yet high heritability rates [2, 3]. Concordance rates increase substantially from 6 % in dizygotic twins to 43 % in monozygotic twins [4]. Children of parents with BD are four times more likely to develop an affective disorder compared to children of parents with no mental disorders [5]. Delayed diagnosis and misdiagnosis are common in BD [6]. Despite evidence for substantial genetic load in the etiology of BD, clinical practice still suffers from the absence of biological markers which could be used in support of the clinical diagnosis.

Endophenotypes are an important subtype of biomarkers that have a clear genetic connection and are more prevalent in patients and in their family members [7]. Exploring multiple endophenotypes to fit in multivariate genetic models for bipolar disorder is an important element in the process of finding diagnostic tools to facilitate early intervention and prevention in BD.

In this review, we aimed to identify common neurocognitive, neuroanatomical, and neurochemical abnormalities that may correspond to vulnerability and resilience factors for BD. Literature on neuroimaging, neurocognition, and biochemical parameters in BD, particularly in populations with high genetic risk for the illness, was reviewed to shed light on the neurobiology of risk for bipolar disorder from an integrative perspective.

## Method

Literature review was completed using keywords “bipolar disorder,” “endophenotype,” “risk,” “relatives,” “neurocognition,” “brain imaging,” and “oxidative stress.” Publications were searched using PubMed, Scopus, Science Direct, and Web of Science electronic databases. Papers published in English, which involved first-degree relatives (FDR-offspring, sibling, co-twins, parents) of a bipolar proband (BD-P) and a healthy control group with or without a patient group, were included. Publications with FDR of schizophrenia probands (SCH-P) in addition to FDR of BD-P, papers on studies modeling for a genetic link to a proposed biological marker, meta-analysis, and systematic reviews were also included. In each respective targeted area, individual studies published after the most recent meta-analysis and/or systematic review were included in the review. This article does not publish original research, animal or human studies, that would need informed consent that should be carried out by the authors.

## Results

### Neurocognition and risk for BD

Genetic influence on measures of various neurocognitive domains has been well documented [8]. Verbal ability, executive functioning, and psychomotor processing speed were shown to be highly heritable in familial BD [9]. A large-scale extended pedigree study suggested impaired processing speed, working memory, and declarative (facial) memory to be candidate endophenotypes for BD [10]. After controlling for demography and current mood symptoms, processing speed was still impaired in BD-P type I and their unaffected FDR, showing its validity as endophenotype to separate BD-P and FDR from healthy controls [11].

In search for potential cognitive endophenotypes, a systematic review and meta-analysis of data from studies on FDR (with or without BD-P) in comparison to healthy controls showed impaired executive function, verbal memory, and verbal working memory [12, 13]. Among executive functions, response inhibition deficits were the most robust candidates followed by impaired verbal memory, sustained attention, and set shifting even after controlling for IQ and age [14].

More recent studies focusing on healthy adolescent offsprings of parents with BD found that young FDR have impairments in processing speed and visual memory [15], cognitive flexibility [16], psychomotor speed, focused attention, verbal attention, phonemic verbal fluency, short-term memory and learning [17], verbal intelligence [18], and significantly slower reaction times on an index of executive attention [19] compared to youth with healthy parents. Likewise, healthy parents of patients with BD-I had significantly worse performance in psychomotor speed, cognitive flexibility, selective attention, response inhibition, and verbal memory [20, 21] than healthy controls. A recent review of conscript, cohort, high-risk, family-based and first-episode mania studies also confirmed that verbal memory and executive function are potential predictors of BD [22].

Recent studies, however, provide further more nuanced evidence specifically with regard to impaired response inhibition and interference control in both adult [23] and adolescent BD-P [24] and their FDR compared to healthy controls. Other studies found that response inhibition was intact despite increased impulsivity and impulsive decision-making in both familial and nonfamilial high-risk groups for BD [25] and interference control was intact in FDR and co-twins of BD-P [26]. Evidence shows significantly worse response inhibition performance in BD-P I with history of psychotic symptoms and their FDR compared to controls [27]. Response inhibition deficit was associated with the process of illness with psychotic features in BD, rather than being a vulnerability marker [28]. On the other hand, impulsivity as measured by BIS-11 (a self-report scale) seems to reveal more consistent signals as a candidate endophenotype both in children, adolescents [29], and adults [25, 30, 31], and as a predictor of onset of BD in reward-sensitive adolescents and young adults [32]. However, specificity of impulsivity to BD is questionable as it shows shared genetic liability with SCH and major depressive disorder [30] and requires further studies. Risk-taking behavior may also be a potential endophenotype and predictor of BD [32, 33].

Recent studies focusing on facial emotion recognition and emotional responsiveness in at-risk relatives compared to healthy controls showed deficits in labeling facial emotion, required significantly more time and more intense emotional information to identify and correctly label face emotions, and were impaired in other aspects of affective response particularly in inhibiting negative valenced stimuli and in having greater response bias toward negatively valenced stimuli [18, 34–39]. Social cognition is another recent area of interest in defining endophenotypes for BD for which theory of mind (ToM) performance has been most commonly considered. BD-FDR performed significantly worse on the verbal but not visual or higher-order ToM tasks compared to healthy controls [40]; their performance was comparable to healthy controls on tasks requiring ToM use and ToM understanding [41].

#### Do cognitive deficits exist before onset of illness in BD?

Systematic review of data from 23 studies on the premorbid cognitive function of people who later developed BD and of BD-P when presenting with their first episode provided evidence that general intelligence is not impaired in the premorbid stage; however, verbal memory, attention, and executive function deficits tend to be present during and after the first episode. Data supports the notion that specific cognitive domain deficits may precede the illness onset in BD [42•]. However, assessment of premorbid intellectual in BD function may yield contradictory findings, depending on whether the assessment is retrospective or prospective [43].

#### Are the neurocognitive markers specific to BD? Role of psychosis

The genetic etiology of BD and SCH overlap substantially [2]. Enhanced susceptibility to interference and reduced inhibition [44] as well as deficits in working memory were reported to be more common in BD patients with psychosis, in SCH patients, and their FDR compared to healthy controls [45]. Severity of premorbid intellectual deficit differs quantitatively between BD and SCH. BD presents significant yet small premorbid intellectual function deficits when assessed retrospectively but not prospectively and moderate cognitive impairment after onset of illness, whereas SCH presents with significant premorbid and large post-onset impairment [43]. It appears that both disorders are associated with impaired visual sustained attention which does not differentiate one condition from another [46].

Further differences between BD and SCH have been observed when examining the association between single nucleotide polymorphisms (SNP) in key risk genes in connection to cognitive tests which are closely linked to prefrontal cortical functioning. The Val66Met polymorphism of the brain-derived neurotrophic factor (BDNF) gene is associated with performance on the Wisconsin Cart Sorting Test in BD but not in SCH, while the reverse is the case for task performance on the N-back working memory task [47]. Among previously identified candidate sets of genes associated with cognitive abilities in SCH or BD visuospatial attention, verbal abilities sets and delayed verbal memory showed the strongest enrichments in BD, whereas color-word interference (cognitive inhibition) test and sets associated with memory learning slope showed enrichment in SCH [48].

Testing SCH-FDR and BD-FDR in comparison to healthy controls on Stroop Color-Word Task and Emotional Stroop Task showed impaired cognitive inhibition in SCH-FDR but emotional bias toward mood-related information in BD-FDR [49]. Assessment of executive functions by using Wisconsin Cart Sorting Test and part A and part B of the Trail Making Task in SCH, BD, and FDR of both groups revealed familial resemblance for both tests in BD families, whereas no resemblance was observed in families with SCH [50]. Using psychosis as a dimension in grouping the participants, a family study revealed a gradient of performance on the working and declarative memory, executive functions, and attention with the poorest being in probands (i.e., SCH-P, BD-P with psychosis), intermediate in FDR of the psychosis spectrum, and highest in the FDR of the nonpsychotic spectrum disorder, supporting the notion that cognitive function in BD and SCH defines a psychosis continuum [51]. Structural equation modeling of cognitive data from 331 twins/siblings showed that illness state and concordance for BD had a modest impact of verbal episodic memory and spatial working memory on the bipolar diathesis; IQ and visual-spatial learning, however, were associated with genetic diathesis to BD and with nonaffective symptomatology, also supporting the notion of psychosis continuum [52].

In an extensive review of studies investigating neurocognitive deficits in premorbid, high-risk and first-episode BD in comparison to outcome studies in SCH, Bora proposed a model where only BD-P who are prone to psychosis may show premorbid neurodevelopmental cognitive deficits similar to SCH. In the absence of psychosis and neurodevelopmental deficits, BD-associated temperamental characteristics set the stage between supranormal premorbid cognition and risk for BP [53••]. Examination of the cognitive profiles of at-risk individuals for BD and BD-P did not appear to support previous suggestions of progressive cognitive decline in BD with illness development [18].

In summary, deficits in executive function, response inhibition, verbal memory/learning, verbal fluency, processing speed, and verbal fluency seem to be promising cognitive markers for risk of developing BD. However, there are limitations in the literature related to the variability of the tests used in measuring the same cognitive domains by different groups, inclusion of varying age groups, nonstandardized definition and use of mixed groups of at-risk individuals (i.e., offspring, siblings, parents), small sample size, and not accounting for the presence of history of psychosis. Such methodological issues cause inconsistencies in the findings and difficulty in interpreting the corresponding functional deficits. Data are still limited on the presence and pattern of premorbid cognitive impairment in the risk population. Findings obtained from cross-sectional studies without controlling for premorbid cognitive impairment may exaggerate the magnitude and misidentify the type of cognitive deficits to be used as markers for risk of BD. Although it may not be specific to BD, the effect of deficits in processing speed on other test performance in patients and to a less extend in the risk groups and controls [54] should be taken into consideration.

### Brain imaging and risk for BD

#### Structural imaging findings

##### Gray matter (GM) abnormalities

In a recent meta-analysis of data from structural and functional imaging studies, the GM volume of individuals at risk for BD did not differ significantly from healthy controls, including regions traditionally associated with BD, such as the striatum, thalamus, amygdala, hippocampus, and pituitary. The results of this meta-analysis challenge the notion that brain morphology can yield endophenotypic markers for BD. The authors also capitalize on the susceptibility of the hippocampus to nongenetic/environmental factors as obstetric complications and stress-induced excessive glucocorticoid exposure. They also draw attention to an association between inconsistent pituitary findings and state-dependent cortisol abnormalities in mood disorders [55]. Assessment of dexamethasone-suppression-CRH test in high-risk individuals who developed an affective disorder in a 10-year follow-up period revealed no premorbid differences in their cortisol response compared to healthy controls [56]. Dysregulated hypothalamo-pituitary-adrenal ( HPA) axis abnormalities in BD can be regarded as a neurobiological scar developing during the course of affective disorders rather than a neuroendocrine vulnerability marker [56]. A later review on studies investigating cortical or subcortical GM abnormalities in BD-FDR shows that findings on various brain regions across studies are inconsistent except for larger insular cortex volumes in adult first-degree relatives and larger right inferior frontal gyrus in BD offspring, in comparison to healthy controls [57].

Recent studies support the above findings with larger inferior frontal gyrus, left insula, smaller cerebellar, and left orbitofrontal gyrus GM volumes being shared both in BD-P and their FDR [58–60], and larger parahippocampal and left dorsolateral prefrontal cortex appeared only in BD-FDR [58, 59].

It is worth remembering that the inferior frontal gyrus has a pivotal role in response inhibition and emotion regulation, both of which have been suggested as candidate endophenotypes for BD whereas the cerebellum has extensive connections to brain areas that are involved in cognition and behavior including the prefrontal cortex, anterior cingulate, and limbic system through cortico-ponto-cerebellar and cerebello-thalamo-cortical pathways. The cerebellum also has a homeostatic role in affect regulation in addition to motor functions [59].

Despite an association between genetic liability for BD and GM volumes in regions of the anterior cingulate cortex, ventral striatum, medial frontal gyrus, right precentral gyrus, right insular cortex, and medial orbital gyrus [57], the absence of evidence for GM abnormalities and contradictory findings [61, 62] across studies in BD-FDR may be due to nongenetic factors such as age, clinical features, medication, duration illness, pubertal stage, and age of onset [63–67]. Increased GM volume or thickness of these regions may be consequent of neuroprotective compensatory mechanisms or abnormal brain maturation due to the maladaptive pruning in at-risk group or may be associated with resilience [59].

The search of a relationship between neuroanatomical changes and genetic risk for BD or SCH showed a specific association between SCH and distributed GM volume loss in the bilateral fronto-striato-thalamic and left lateral temporal regions and enlarged lateral ventricles; genetic risk for BD was specifically associated with GM deficits in the right anterior cingulate gyrus and ventral striatum [55].

##### White matter (WM) abnormalities

There is a limited number of diffusion tensor imaging (DTI) studies of unaffected FDR of BD-P. Some studies found abnormalities in the superior longitudinal fasciculus, inferior longitudinal fasciculus, corpus callosum, right uncinate fasciculus, right inferior fronto-occipital fasciculus, right anterior limb of internal capsule, and thalamic radiation in both BD-P and BD-FDR [68•, 69], while two studies did not find any abnormalities in older relative groups compared with controls [70, 71]. A population-based study showed abnormalities in similar white matter tracts in adolescents with subthreshold bipolar symptoms [72]. These tracts connect regions implicated in the identification and regulation of emotion, attention, impulsivity, response inhibition, set shifting, and risk-taking [68•]. Decreased WM volume is highly associated with genetic risk and familiality in BD [70–74]. However, the results are neither consistent nor robust enough to indicate replicable WM abnormalities in BD-FDR, thus not supportive of the WM abnormalities as an endophenotype of BD, whereas more WM abnormalities in SCH conform better to the concept of endophenotype [68•, 73].

#### Functional neuroimaging

Recent years have witnessed the publication of a sizeable number of task and resting state fMRI in BD-P and their FDR in comparison to healthy controls.

##### Resting state

Resting state connectivity between the frontal cortex and basal ganglia or limbic/paralimbic regions was shown to be altered in a nonspecific fashion in unaffected BD-FDR [75•]. Solé-Padullés et al. found no connection differences between any regions in BD-FDR compared with SCH-FDR and healthy controls [76].

##### Cognitive task—fMRI studies

When reviewing the relevant studies, we found that patients and their parents show activation differences in the frontal cortex, insula, amygdala, parietal cortex, and cingulate cortex as well as connectivity defects between these regions [55, 75•]. Hyperactivation of inferior frontal gyrus (including ventrolateral prefrontal cortex and orbitofrontal cortex) and hypoactivation of insula, amygdala, basal ganglions, and limbic system, which were signified in at-risk group, are interpreted as compensatory mechanisms [75•, 77, 78]. An fMRI study found differential effects of the DISC1 Leu607Phe polymorphism on the left pre/postcentral gyrus, extending to inferior frontal gyrus in FDR of BD and SCH during language task [79]. However, relatively small sample sizes of the studies limit the generalizability of the findings.

##### Emotional task—fMRI studies

Studies which investigated neural substrate of emotional dysregulation in risk groups for BD showed altered activity in insula and ventrolateral prefrontal cortex, dysfunctional connectivity in orbitofrontal cortex-amygdala, and impairment in downregulation of amygdala [75•, 77, 80–83]. Breakspear et al. showed impaired hierarchical model (dorsolateral prefrontal cortex-inferior frontal gyrus-anterior cingulate cortex) and reduced activity of inferior frontal gyrus in BD relatives [84]. In the risk group, the change produced by the negative affect in the brain regions was more evident than the positive affect [81, 83]. This is an important finding, yielding a new research area in the light of the fact that response to positive affect is more sensitive to environmental factors and that it could easily be lost compared to the control group [81, 83].

In summary, fMRI studies present alterations in the activity of the same regions involved in the pathophysiology of BD, namely the inferior frontal gyrus, medial prefrontal cortex, and limbic area particularly in the amygdala, in unaffected BD-FDR, in comparison to healthy controls.

#### Genetics and white matter neuroimaging in the risk for BD

Studies combining genetics and neuroimaging demonstrated association between decreased WM in BD-FDR and disrupted NRG1-ErbB4, calcium signaling (CACNA1C), phosphatidylinositol, and CAMs pathways [74, 85]. These pathways relate to WM development, neuronal plasticity, regulation of neurotransmitter release, and cell adhesion [74]. Another study reported an association between FA reduction in the WM tracts which are involved in the pathophysiology of BD and higher polygenic risk scores in affected but not in unaffected relatives [86], which suggest that WM abnormalities are closely linked to expression of psychopathology rather than genetic risk per se.

Overall, there are discrepancies in the results from both structural and functional imaging studies despite promising findings identifying risk markers for BD. Different imaging techniques that had been applied, heterogeneous clinical and demographical profiles of the participants, small sample size, variability of the tasks, heterogeneity of the definition of the risk groups (offspring vs. siblings vs. parents, vs. mixed group of FDR), presence of subsyndromal symptoms in the risk groups in some studies as well as variability in age of the participants contribute to difficulties in identifying a specific pattern of alterations for individuals at risk. Also, the effects of environmental factors and the association between clinical features and MRI findings are not well known. The absence of fMRI studies investigating social cognition, risk-taking, and response inhibition, is also noteworthy.

### Inflammation, oxidation, neurotrophins, and other mediators and risk for BD

Exploration of the inflammatory processes on the neuronal function of risk groups is important for a better understanding of the molecular basis of risk for BD as accumulating data implicate these processes in the pathogenesis of BD.

Among several molecules which are suggested to be involved in the pathogenesis of BD and are known to be involved in inflammation (interleukin 1, interleukin 6 (IL-6), interleukin 10, interleukin 17, interferon gamma), oxidative stress (thiobarbituric acid reactive substances, protein carbonyl content), and neurotrophins (BDNF), only IL-6 levels have been found to be significantly higher in BD-P compared to BD-FDR [87]. However, increased IL-6 and BDNF plasma levels have been reported in BD offspring compared with healthy controls. High-risk offspring that appeared to have prodromal symptoms presented with higher plasma levels of IL-6 and BDNF than high-risk offspring that appeared asymptomatic or mildly symptomatic [88].

In a prospective follow-up, BD offspring showed increased proinflammatory gene expression in monocytes during adolescence, but not in adulthood [89]. Specifically, in that study, BD offspring had persistent monocyte activation during adolescence and early adulthood as shown by increased cytokine pentraxin-related protein (PTX3) levels and T-regulatory cells and decreased effector T cells (Th1 and Th17). Despite decreased serum levels of BDNF, normal levels of chemokine (C-C motif) ligand 2 (CCL2), and S100 calcium-binding protein B (S100B) during adolescence, BD offspring showed increased levels of CCL2, BDNF, and S100B in adulthood [89]. These findings suggest an abnormal neuroimmune state in BD offspring, which followed a dynamic course from adolescence into adulthood.

Most recently, plasma levels of lipid peroxidation (lipid hydroperoxide and 4-hydroxy-2-nonenal, 8-isoprostane), protein oxidation (protein carbonyls), and inflammation (interleukin 1, interleukin 6, interleukin 10, interferon gamma, TNF alfa) were assessed in four groups of adolescents (9–20 years of age), consisting of high-risk offspring, ultrahigh-risk offspring, first-episode BD patients, and healthy controls [90]. The levels of lipid hydroperoxide, an early stage lipid peroxidation marker, showed a decreasing trend along the spectrum of risk for BD-I, while there was no difference in the late stage lipid peroxidation markers (4-hydroxy-2-nonenal, 8-isoprostane), protein carbonyls, and inflammatory markers among groups [90].

Serum BDNF levels were found to be decreased [91] or unchanged [92, 93] in BD-P and BD-FDR compared to healthy controls. Duffy et al. reported that the BDNF genotype significantly moderates the association between high-risk status for both gene expression and protein levels in BD offspring [88]. Correspondingly, anxiety symptoms were associated with the BDNF risk genotype only in BD offspring but not in healthy controls, and BD offspring with the val/val genotype showed higher anxiety symptoms than BD offspring with other genotypes [94].

Ferensztajn et al. reported higher BDNF and matrix metalloproteinase-9 levels and lower IL-6 levels in the offspring of BD-P who were excellent lithium responders compared to the offspring of BD-P who were lithium nonresponders [95].

Comparison of biomarkers related to oxidative stress [8-hydroxy-2′-deoxyguanosine (8-OHdG), mitochondrial complex 1 activation, and glutathione peroxidase activities] and global DNA methylation (5-methylcytosine) between lithium responder BD-P, BD-FDR and healthy controls showed that BD-FDR have decreased global methylation, increased glutathione peroxidase activity, and no change in 8-OHdG or in mitochondrial complex 1 activity [96].

These results show that risk groups for BD present with altered immune and neurochemical modulation. However, the findings are preliminary, and studies on well-defined and clinically homogeneous risk groups, particularly in prospective design to understand the risk and defense mechanisms, are needed.

## Conclusion and future directions

Prospective long-term follow-up studies using multimodal (i.e., combination of imaging, cognitive, neurochemical, and genetic assessment of the participants) and standardized techniques (i.e., the same set of cognitive tasks per domain) in well-defined at-risk populations, controlling for age and gender distribution as well as for the presence of symptoms, are needed for better understanding of the neurobiology of risk for BD [97]. Operationalized criteria for defining risk and resilience markers would also assist in improving our understanding of the complex changes observed in patients and their relatives. This would also foster further research into disambiguating compensatory from pathological processes. It is unclear whether disease-specific biomarkers can indeed be identified as most indications point to significant overlap between disorders which has generally motivated a trans-diagnostic approach to psychiatric research. Another unmet need is the information on interaction between immune and neurochemical alterations and cognitive and structural/functional changes. Studies exploring the associations between neurochemical, cognitive, and imaging are needed. Multicenter, population-based studies with large sample size allowing for homogeneous subgroup analysis (i.e., relatives of BD type I vs. type II, psychotic vs. nonpsychotic, offspring vs. sibling, symptomatic vs. asymptomatic, adolescent vs. adult; treatment responder vs. nonresponder) searching for cognitive, imaging, and neurochemical modulatory markers for risk for BD in an integrative way will immensely contribute to the field.

## Highlights of the review

The review includes data on cognitive functions, structural and functional imaging, and neurochemical modulation as potential markers for risk and resilience for BD in an integrative way. The most recent data in each respective field has been included in the review besides meta-analysis and systematic reviews.

Despite inconsistencies, compiling data reveals cognitive and imaging markers for risk and to a less extent resilience of BD. Findings on neural modulation markers are preliminary and require further studies. Methodological issues causing obstacle in interpretation of the existing data have been considered.

## Abbreviations

BD: Bipolar disorder
BDNF: Brain-derived neurotrophic factor
BD-P: Bipolar disorder probands
CACNA1C: Calcium voltage-gated channel subunit alpha1
CCL2: Chemokine (C-C motif) ligand 2
DISC1: Disrupted in schizophrenia 1
FDR: First-degree relative (including offspring, sibling, co-twins, parents)
fMRI: Functional magnetic resonance imaging
GM: Gray matter
IL-6: Interleukin 6
8-OHdG: 8-Hydroxy-2′-deoxyguanosine
PTX3: Pentraxin-related protein
S100B: S100 calcium-binding protein b
SCH: Schizophrenia
SCH-P: Schizophrenia probands
ToM: Theory of mind
WM: White matter
